# Tumor-associated neutrophils activated by tumor-derived CCL20 (C-C motif chemokine ligand 20) promote T cell immunosuppression via programmed death-ligand 1 (PD-L1) in breast cancer

**DOI:** 10.1080/21655979.2021.1977102

**Published:** 2021-09-14

**Authors:** Louis Boafo Kwantwi, Shujing Wang, Wenjun Zhang, Weidong Peng, Zeyu Cai, Youjing Sheng, Han Xiao, Xian Wang, Qiang Wu

**Affiliations:** a Department of Pathology, School of Basic Medical Science, Anhui Medical University, Hefei, PR China; b Department of Immunology, School of Basic Medical Science, Anhui Medical University, Hefei, PR China; c Department of Pathology, The First Affiliated Hospital of Anhui Medical University, Hefei, PR China; d Department of Pathology, The Second Affiliated Hospital of Anhui Medical University, Hefei, PR China

**Keywords:** Tumor-associated neutrophils, CCL20, PD-L1, immunosuppressive, breast cancer

## Abstract

Breast cancer is the leading cause of cancer-related death among women despite the significant improvement in diagnosis and treatment. Tumor-associated neutrophils have been shown to suppress antitumor functions of the host. However, how breast cancer tumor microenvironment influences the phenotype and functions of neutrophils to potentiate T cell immunosuppression is unknown. Herein, neutrophils isolated from peripheral blood of healthy donors were treated with supernatants from breast cancer cell lines or recombinant human CCL20. PD-L1 expression on neutrophils was then evaluated by immunofluorescence and flow cytometry. Neutrophils and Jurkat T cells were cocultured to evaluate the effect of tumor-associated neutrophils on T cell functions. Finally, immunohistochemical staining was performed to evaluate the clinical relevance of neutrophils infiltrating breast tumor tissues. Tumor-derived CCL20 activated and upregulated PD-L1 expression on neutrophils. A significant positive correlation was found between CCL20 and CD66b+ neutrophils in tumor tissues. Through in vitro experiment, tumor-associated neutrophils (TANs) effectively suppressed T cell immunity which was reversed upon PD-L1 blockade.

Moreover, a high density of TANs was associated with short disease free survival in breast cancer patients. Furthermore, receiver operating curve showed that the density of TANs could accurately predict disease-free survival in breast cancer patients. Our findings suggest that targeting TANs via CCL20 immunosuppressive pathway may be a novel therapeutic strategy for breast cancer treatment.

## 1. Introduction

Breast cancer continues to be the commonly diagnosed and the leading cause of cancer-related death among women [1]. Regardless of the substantial advances made in diagnosis, approximately 5–10% of patients show distant metastasis at the time of diagnosis [2]. Even with the improvements in standard chemotherapy and hormonal therapy, recurrence and metastasis occur, contributing to 90% of breast cancer-related deaths [2,3]. In clinical settings, the prognostic assessment of breast cancer patients is based on a set of widely validated factors such as tumor stage, lymph nodes status, histological grade of differentiation and molecular type [4,5]. However, due to the heterogeneous nature of the disease, patients with similar prognostic features have different clinical outcomes. Hence, a deeper understanding of the pathogenesis of breast cancer is needed as we seek to identify new and effective targets for breast cancer treatment.

A substantial body of evidence has linked the tumor microenvironment in tumor development [6,7]. This supportive role results from the infiltration of the tumor stroma by several immune and inflammatory cells, including neutrophils, macrophages and mast cells [8,9]. Neutrophils are critical immune cells in the host defense mechanism during infections [10]. Nevertheless, accumulating evidence suggests that neutrophils contribute to the pathogenesis of several diseases, including cancers. It has been proven that neutrophils infiltrated into the tumor microenvironment exert antitumor and pro-tumor functions [11–14]. Tumor-associated neutrophils (TANs) participate in tumor progression by promoting tumor growth, invasion, angiogenesis and metastasis [15]. Besides these tumor-promoting mechanisms, TANs have been shown to impair the antitumor immune response by expressing immunosuppressive molecules, such as programmed cell death ligand 1(PD-L1) [16,17]. Despite the increasing knowledge in this area, how the tumor microenvironment influences PD-L1 expression on TANs in breast cancer remains unknown.

CCL20, the sole receptor of CCR6, plays a direct role in the progression of several solid tumors, including breast cancer [18–20]. Recently, studies have shown that CCL20 promotes tumor progression by remodeling the functions and phenotype of immune cells infiltrated into the tumor microenvironment [21,22]. Therefore, we hypothesized that CCL20 might have a biological effect on neutrophils infiltrated into the tumor microenvironment. Herein, we sought to elucidate the interplay between tumor-derived CCL20 and neutrophils, emphasizing how such interactions suppress antitumor immunity of the host via PD-L1+ neutrophils.

## 2. Materials and methods

### 2.1 Patients and specimens

Formalin-fixed-paraffin-embedded tissue specimens were obtained from 102 breast cancer patients who underwent surgical operation from 2014 to 2019 at The First Affiliated Hospital of Anhui Medical University. Patients included in this study had not received any preoperative adjuvant therapy.

### 2.2 Cell cultures and enzyme-linked immunosorbent assay (ELISA)

MDA-MB-231, MCF-7 and T47D cell lines were obtained from Shanghai Cell Bank. MDA-MB-231, MCF-7 and T47D cells were cultured in Leibovitz’s-15 (L-15), Rosewell Park Memorial Institute 1640 medium (RPMI 1640) and Dulbecco’s Modified Eagle Medium (DMEM) respectively at 37°C with 5% CO_2_ where appropriate [23]. These culture media were supplemented with 10% fetal bovine serum (FBS) and 1% penicillin-streptomycin. Jurkat T cell lines were maintained in RPMI1640 culture medium supplemented with 10% FBS and 1% penicillin-streptomycin at 37°C with 5% CO_2_.

The concentration of CCL20 in supernatants from MDA-MB-231, MCF-7 and T47D cell lines was determined using ELISA (ABclonal, 86 Cummings Park Dr, Woburn, MA 01801, USA).

### 2.3 Neutrophil isolation and stimulation

For each experiment, neutrophils were isolated from three healthy male volunteers after obtaining informed consent.

Peripheral blood was drawn into ethylenediaminetetraacetic acid (EDTA) tubes and centrifuged at 250 *g* for 10 minutes. Once centrifugation was complete, supernatants were discarded, and sodium chloride (NaCl) was added to the blood. Ficoll was drawn into a centrifuge tube and the blood was carefully layered on top of the ficoll. This suspension was centrifuged at 450 *g* for 25 minutes, after which the supernatants were discarded. Subsequently, red lysing buffer was added to the blood and kept on ice for 10 minutes. The mixture was centrifuged at 1500 rpm for 5 minutes, after which the supernatants were discarded. Once the lysing step was complete, cells were washed with phosphate buffer saline (PBS) and centrifuged at 1500 rpm for 5 minutes.

Neutrophils (2 × 10^5^) were stimulated with recombinant human CCL20 (PEPROTECH Rocky Hill, NJ 08553, USA, Cat No. 300-29A) and supernatants from MDA-MB-231, MCF-7 and T47D. For blockade experiments, CCL20 neutralizing antibody (R&D Systems, Minneapolis, MN, USA, Cat No. AF360-SP) was added to the coculture of neutrophils and supernatants from breast cancer cell lines.

### 2.4 Flow cytometry assay

## Neutrophil survival assay

Neutrophils were stimulated with recombinant human CCL20 and supernatants from breast cancer cell lines for 10 hours. After 10 hours of stimulation, the viability of neutrophils was quantified using Annexin V Apoptosis detection kits (Best Bio).

## PD-L1 evaluation

Stimulated neutrophils were washed three times with PBS and stained with 5ul of PD-L1 (eBiocience^TM^ Cat No. 12-5983-42) on ice for 30 min. PD-L1 was evaluated using the Cytoflex flow cytometry machine and analyzed using CytExpert 2.4.

## Neutrophil- T cell coculture system

Jurkat T cells (5 × 10^6^) were labeled in 1 ml of PBS containing 5 µM of carboxyfluorescein succinimidyl ester (CFSE, Biolegend, Cat No. 423801) for 20 minutes and seeded at 200,000 cells in a 24 well plate. Stimulated neutrophils (TANs, 2 × 10^5^) were cocultured with Jurkat cells (2 × 10^5^) in 1 ml of RPMI 1640 containing 2ul phytohemagglutinin-L antibody (eBiocience^TM^, Cat No. 00-4977-93). For blockade study, TANs were cocultured with Jurkat T cells in the presence of neutralizing antibody against PD-L1 (D-PPA 1 PD-1/PD-L1 inhibitor, TOCRIS, Minnesota, USA, Cat No. 6515).

Jurkat T cells cocultured with TANs were harvested and stained with anti-human CD3 (BioLegend, Clone HIT3a). For IFN-γ (BioLegend, Clone B27) determination, intracellular cytokine staining was performed by fixation and permeabilization. Following this, the content of CFSE and production of IFN-γ on CD3 cells were evaluated using BD flow cytometry machine.

### 2.5 Immunofluorescence staining

Immunofluorescence staining was performed following previous report [24]. Neutrophils were smeared onto a poly lysine-treated sterile glass slide. Cells were fixed using 200 µl of 4% paraformaldehyde for 1 hour and then permeabilized with 0.5% Triton X-100 in PBS for 15 minutes. Following this, cells were blocked for 30 minutes by a blocking buffer containing 1% bovine serum albumin (BSA) in PBS. The cells were then incubated with anti-PD-L1 antibody (dilution 1:500, Ventana, Roche, Clone SP142) in PBS containing 1% BSA for overnight at 4°C. Samples were washed three times and incubated with Alexa Fluor-conjugated secondary antibody (dilution 1:500, Abcam) for 1 hour. Finally, samples were incubated with 4,6-diamino-2-phenylindole (DAPI) (Beyotime, Shanghai, China) in the dark for 5 minutes. PD-L1 expression was observed using laser scanning confocal microscope 880.

### 2.6 Immunohistochemistry (IHC)

Immunohistochemical staining was performed as previously described [25]. Briefly, formalin-fixed-paraffin-embedded tissues were cut into 4 μm sections and incubated on slides. The sections were deparaffinized in xylene and rehydrated in ethanol solution of decreasing concentrations (100%, 95% and 75%). Antigen retrieval was performed by incubating slides in 0.01 mol/L sodium citrate buffer (pH 6.0) for 10 minutes. Slides were incubated with primary antibodies against CCL20 (Proeintech, Cat No. 26527-1-AP), CD3 (Ventana, Roche, Lot F16525), CD66b (BD Pharmingen^TM^, Clone No G10F5), IFN-γ (Bioss Antibodies, Cat No. bs-0481 R) and PD-L1 (Ventana, Roche, Clone SP142) at 4°C for overnight. The slides were then incubated with goat-anti mouse and rabbit secondary antibody for 30 minutes at room temperature. This was followed by the addition of 3, 3ʹ-diaminobenzidine tetrahydrochloride (DAB) for 1–5 minutes in the dark. Slides were incubated with hematoxylin for 2 minutes, and the images were observed using Olympus CX33 microscope.

### 2.7 IHC evaluation

The densities of CD3 T cells and CD66b+ neutrophils in tumor tissues were assessed as previously reported [26]. Briefly, CD66+ neutrophils and CD3 T cells were counted from 10 random microscopic sites (×400). The average count of the entire 102 sections was used to divide CD66b into high and low groups. CCL20 staining intensity was graded on a scale of 0–3 as follows: 0, absence of staining; 1, weak staining; 2, moderate staining; 3, strong staining. Positive tumor cells were similarly scored on a scale of 0–4 as follows: 0, absence of tumor cells; 1, 1–25% positive tumor cells; 2, 26–50% positive tumor cells; 3, 50–75% positive tumor cells; 4, >75% tumor cells. The IHC scores (0–12) were obtained by multiplying the staining intensity by the percentage scores. Based on previous report [27], PD-L1 expression on immune cells was evaluated as follows: IC3 (≥10%), IC2 (≥5 < 10%), IC1 (≥1% and <5), and IC0 (<1%). The INF-γ expression on immune cells was scored on a scale of 1–10.

### 2.8 Data analysis

Correlation analyses were performed using Spearman where appropriate. Student’s- *t*-test was used to analyze the difference between two groups. All data were analyzed using two-tailed test and *P* < 0.05 was considered statistically significant. All statistical analyses were performed using SPSS 21.0 software (SPSSInc, Chicago, IL) and GraphPad Prism 5.0 software package (GraphPad Software, Inc., San Diego, USA).

## 3. Results

Soluble factors secreted by tumor cells have been shown to activate neutrophils infiltrating the tumor microenvironment. Therefore, we hypothesize that CCL20 secreted by breast cancer cells can promote immunosuppressive functions of neutrophils. This study was aimed at assessing how tumor-derived CCL20 activated neutrophils into TANs and how these TANs inhibited T cell immunity through PD-L1 pathway.

### 3.1 Tumor-derived CCL20 activated and induced PD-L1 expression on neutrophils

**Figure 1.**
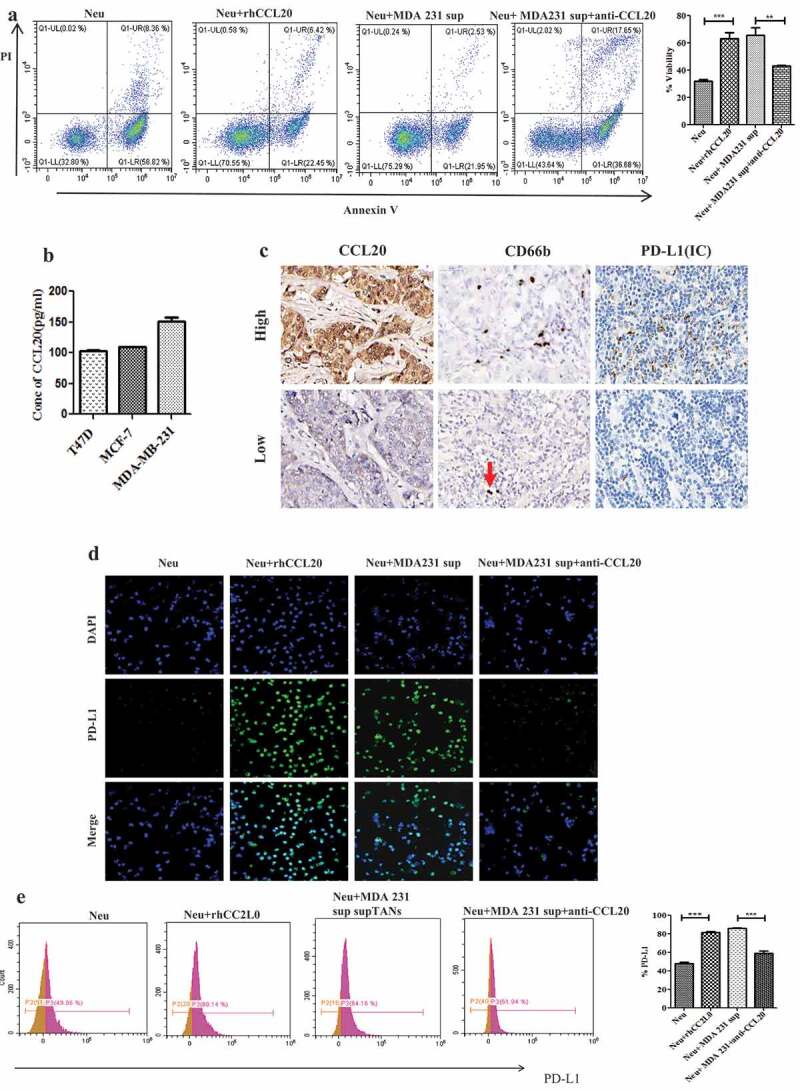
Tumor-derived CCL20 activated and induced PD-L1 expression on neutrophils. (a) Viability of neutrophils treated with recombinant human CCL20 or supernatant from MDA231cell lines. (b) CCL20 expression as determined by ELISA in cell culture supernatant from breast cancer cell lines. (c) The expression of CCL20, CD66b and PD-L1 in breast cancer tissues (Immunohistochemistry, ×400). (**d** and **e)** PD-L1 expression on neutrophils cultured with rhCCL20 and MDA-MB-231 supernatant in the presence of CCL20 neutralizing antibody as determined by immunofluorescence and flow cytometry, respectively

To evaluate the functions of tumor-derived CCL20 on neutrophils, we determined the viability and expression of PD-L1 on neutrophils treated with supernatants from breast cancer cell lines and recombinant human CCL20 (rhCCL20). As shown in Figure 1a, the viability of neutrophils treated with rhCCL20 or supernatant from MDA-MB-231 cell line was significantly (*P* < 0.0001) higher than neutrophils cultured alone. To further confirm the role of tumor-derived CCL20 in the activation of neutrophils, CCL20 neutralizing antibody was added to the coculture of neutrophils and supernatant from MDA-MB-231 cell line. It was observed that the viability of neutrophils was significantly decreased *(P *< 0.001), suggesting that tumor-derived CCL20 can activate neutrophils infiltrating the tumor microenvironment.

Evidence has shown that tumor-derived factors can induce PD-L1 expression on neutrophils. Hence, we wanted to determine whether tumor-derived CCL20 can induce PD-L1 expression on neutrophils in breast cancer. The levels of CCL20 in the cell culture supernatants from MDA-MB-231, MCF-7 and T47D cell lines (Figure 1b) were evaluated. Due to the presence of CCL20 in these supernatants, neutrophils were cocultured with these supernatants for PD-L1 evaluation. Immunofluorescence results showed a higher expression of PD-L1 on neutrophils treated with rhCCL20 or supernatants from MDA-MB-231 (Figure 1d), MCF-7 (Fig S1) and T47D (Fig S1) cell lines than neutrophils cultured alone. However, addition of CCL20 neutralizing antibody to the coculture of neutrophils and supernatants from these cell lines downregulated PD-L1 expression on neutrophils. Flow cytometry results confirmed a significantly (*P* < 0.001) higher expression of PD-L1 on neutrophils treated with rhCCL20 or supernatants from (MDA-MB-231, Figure 1d), (MCF-7 Fig S2) and (T47D Fig S2) (Figure 1d) than neutrophils cultured alone. However, addition of CCL20 neutralizing antibody to the coculture of neutrophils and supernatants from these cell lines significantly (*P* < 0.001) downregulated PD-L1 expression on neutrophils.

To establish a connection between CCL20, TANs and PD-L1, immunohistochemistry was performed to evaluate the expression of these markers in 102 breast cancer tumor samples. The analysis showed that the expression of CCL20 was positively correlated to the density of CD66b+ neutrophils (Figure 1c, *r* = 0.594, *P* < 0.001) and PD-L1 expression on immune cells (Figure 1c, *r* = 0.409, *P* < 0.001). Further, the density of CD66b+ neutrophils showed a positive correlation to PD-L1 expression on immune cells (Figure 1c, *r* = 0.406, *P* < 0.001). This suggested a possible relationship between these three markers in breast cancer tumor tissues.

Collectively, these results indicated that tumor-derived CCL20 activated and induced PD-L1 expression on neutrophils.

### 3.2 TANs promote T cell immunosuppression through PD-L1 in breast cancer

**Figure 2.**
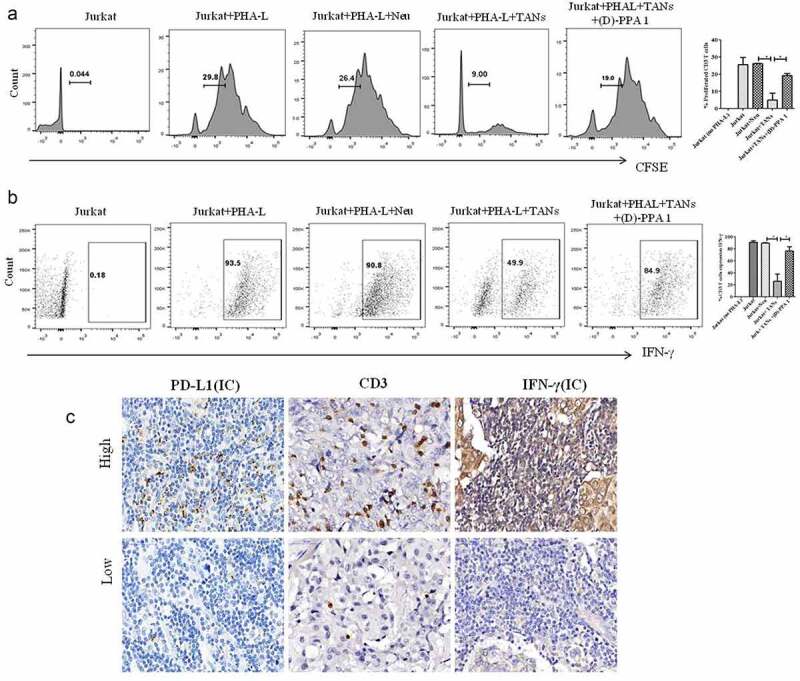
TANs promote immunosuppression via PD-L1 in breast cancer. (a) Proliferation of CFSE-labeled T cells co-cultured with neutrophils (Neu) and TANs as examined by flow cytometry. (b) The production of IFN-γ from T cells co-cultured with Neu and TANs, as examined by flow cytometry. (c) The expression of PD-L1, CD3 and IFN-γ in breast cancer tissues (Immunohistochemistry, ×400)

TANs have been demonstrated to promote immunosuppression via PD-L1 in several cancer types. Our next objective was to evaluate the effect of TANs on T cell functions. Jurkat T cells were cocultured with neutrophils treated with supernatant from breast cancer cell lines. Interestingly, TANs significantly suppressed proliferation (Figure 2a, *P* = 0.0057) and production of IFN-γ (Figure 2b, *P = *0.0256) on CD3 T cells. To determine the involvement of PD-L1+ neutrophils in T cell immunosuppression, PD-L1 inhibitor (D-PPA 1) was added to the coculture of TANs and Jurkat T cells. Notably, the blockade of PD-L1 significantly restored both proliferation (*P* = 0.0037) and production of IFN-γ (*P* = 0.0229) on CD3 T cells. Collectively, these findings indicated that TANs but not neutrophils might promote T cell immunosuppression through PD-L1 in breast cancer.

**Figure 3.**
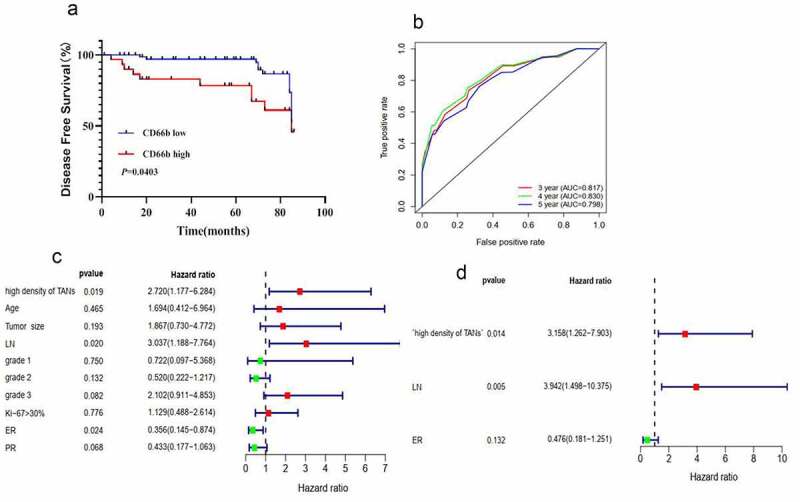
CD66b+ neutrophils predict poor survival in breast cancer patients. (a) High density of CD66b predicts short disease-free survival in breast cancer patients. (b) ROC curve of the predictive value of CD66b . (c) Uni-Cox regression analyses of makers . (d) Multi-Cox regression analyses of markers

We also performed immunohistochemical staining to evaluate the relationship between CD3, IFN-γ and PD-L1 expression on immune cells (ICs). PD-L1 (ICs) correlated negatively to the density of CD3 T cells (*r* = −0.315, *P* = 0.037) and the expression of IFN-γ (ICs) (*r* = −0.407, *P* = 0.008) (Figure 3c). Also, the density of CD66b+ neutrophils showed a negative correlation to CD3 T cells (*r* = −0.427, *P* < 0.001) and IFN-γ (ICs) (*r* = −0.451, *P* < 0.001).

### 3.3 CD66b+ neutrophils predict poor survival in breast cancer patients

Given the ability of TANs to promote T cell immunosuppression, we evaluated the clinical relevance of TANs in breast cancer. Survival analysis showed that high density of TANs was associated with short disease-free survival in breast cancer patients (Figure 3a). From the receiver operating curve (ROC), the density of TANs accurately predicted the disease-free survival for 3–5 years (Figure 3b). Univariate (Figure 3c) and multivariate (Figure 3c) Cox regression analyses revealed that the density of TANs was an independent prognostic factor for disease-free survival in breast cancer patients. Unsurprisingly, a high density of TANs was observed in triple-negative breast cancer patients than the other subtypes of breast cancer (S3).

## 4. Discussion

Neutrophils, the first respondents to tissues damage, have been demonstrated to play crucial roles in several solid tumors. Emerging evidence has documented both protumor [28,29] and antitumor [12,30] functions of neutrophils, highlighting our lack of detailed knowledge about their diversity and functions in the tumor microenvironment. It has been suggested that the plasticity in phenotype and function of neutrophils is mediated by the distinct signals in the tumor microenvironment [10,31]. Therefore, deciphering the exact role of TANs would be particularly useful in the development of TANs targeted therapy. In this research, we showed for the first time how tumor-derived CCL20 induces immunosuppressive neutrophils to inhibit T cell immunity in breast cancer.

The predictive value of neutrophils infiltrating the tumor microenvironment has been reported in several cancer types. The findings from this present demonstrated that high density of CD66b+ neutrophils is not only associated with short disease-free survival but can be used as a marker to predict breast cancer progression. Consistent with our findings, a high density of CD66b+ neutrophils has been reported to be associated with poor prognostic outcomes in germ [26] colorectal [32] and cervical [33] cancer patients.

PD-L1 expression on neutrophils is regulated by several factors such as inflammation, infections and cancers [34]. In cancers, studies have reported that tumor-derived factors such as granulocyte-macrophage colony-stimulating factor (GM-CSF) [16], interleukin 6 (IL-6) [35], long non-coding RNA HOXA transcript at the distal tip (HOTTIP) [17] and high-mobility group box-1 (HMGB1) [36] can induce PD-L1 expression on neutrophils. Herein, our in vitro study proved that both recombinant human CCL20 and tumor-derived CCL20 can induce PD-L1 expression on neutrophils, thus revealing tumor-derived CCL20 as a regulator of PD-L1 expression. Furthermore, immunohistochemical analysis revealed a positive correlation between CCL20 and CD66b+ neutrophils in tumor tissues, which further supports a possible interaction between CCL20 and TANs in tumor microenvironment.

Mechanisms leading to suppression of host antitumor immunity are widely known to be a hallmark of cancer. It has been suggested that the interaction between PD-L1 and PD-1 is a key mechanism responsible for immunosuppression in cancers. In principle, the binding of PD-L1 to PD-1 effectively suppresses the proliferation and activation of T cells [37,38]. This study revealed that PD-L1 expression on TANs effectively inhibited proliferation and IFN-γ production on T cells. This finding is consistent with other reports in gastric and hepatocellular cancers where TANs displayed a remarkable ability to suppress T cell immune response via PD-L1 expression [16,35,39]. Taken together, our report suggests that the activation of neutrophils by tumor-derived CCL20 is necessary to suppress and impair the antitumor immunity. This reveals new insight into the role of CCL20 in breast cancer pathogenesis.

Furthermore, PD-L1 expression on immune cells (ICs) correlated negatively with the density of CD3 T cells and IFN-γ (ICs), which further supports the role of PD-L1 in tumor related-immunosuppression. Concordance with these findings, PD-L1 expression on tumor-infiltrating immune cells has been noted to play a key role in suppressing the antitumor immunity independent of PD-L1 expression on tumor cells. More importantly, PD-L1 expression on immune cells was an indicator of immunosuppression in tumors [40].

## 5. Conclusion

**Figure 4.**
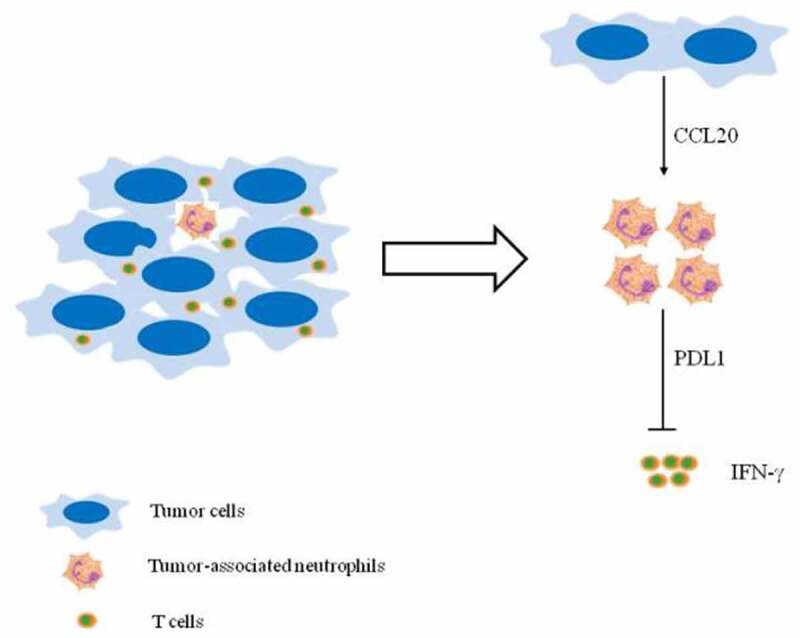
**(Graphical Abstract). Proposed mechanism depicting CCL20 – mediated immunosuppression via PD-L1+ neutrophils**. Tumor-derived CCL20 activates and induces PD-L1 expression on neutrophils. These neutrophils inhibited T cell functions via PD-L1 in breast cancer

Based on our findings, we propose a model involving T cell immunosuppression in breast cancer (Figure 4). First, CCL20 secreted by breast cancer cells activates and induces PD-L1 expression on neutrophils. Second, these activated neutrophils exert protumor functions by suppressing T cell functions via PD-L1. Hence, targeting TANs via CCL20 may be a novel therapeutic strategy for breast cancer treatment. Despite the novelty of our findings, further in vivo studies will be required to explain the underlying mechanism by which CCL20-TANs-PD-L1 network influences immunosuppression in breast cancer.

## Supplementary Material

Supplemental Material

## Research highlights

1. Tumor-derived CCL20 activated and induced PD-L1 expression on neutrophils.
2. Tumor-associated neutrophils promoted T cell immunosuppression via PD-L1 in breast cancer.
3. High density of tumor-associated neutrophils was associated with short disease-free survival in breast cancer patients.
