# Supplementary figures and images for: Tumor-associated neutrophils activated by tumor-derived CCL20 (C-C motif chemokine ligand 20) promote T cell immunosuppression via programmed death-ligand 1 (PD-L1) in breast cancer
Source: Bioengineered. 2021 Sep 14;12(1):6996–7006. doi: 10.1080/21655979.2021.1977102 (PMC8806641; doi:10.1080/21655979.2021.1977102)

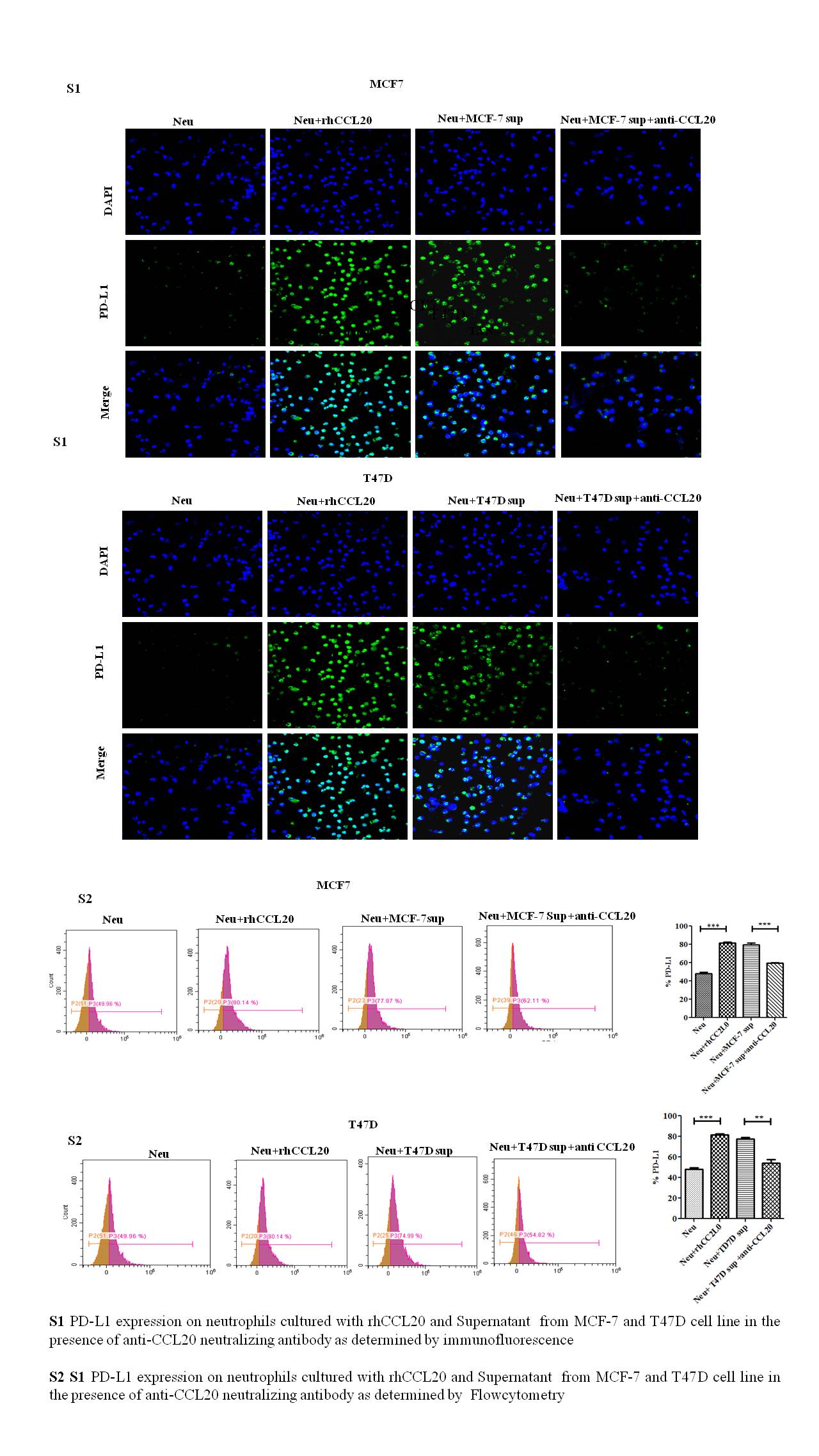

Supplement: Supplemental Material [file KBIE_A_1977102_SM2078.jpg]
